# Health System Redesign to Shift to Hospital Delivery for Maternal and Newborn Survival: Feasibility Assessment in Kakamega County, Kenya

**DOI:** 10.9745/GHSP-D-20-00684

**Published:** 2021-12-31

**Authors:** Kojo Nimako, Anna Gage, Caroline Benski, Sanam Roder-DeWan, Khatra Ali, Charles Kandie, Aisha Mohamed, Hellen Odeny, Micky Oloo, John Tolo Boston Otieno, Maximilla Wanzala, Rachel Okumu, Margaret E. Kruk

**Affiliations:** a Department of Global Health and Population, Harvard T.H. Chan School of Public Health, Boston, MA, USA.; b Ifakara Health Institute, Dar es Salaam, Tanzania.; c Kenya Council of Governors, Nairobi, Kenya.; d Kenya Ministry of Health, Nairobi, Kenya.; e Kakamega County Department of Health, Kakamega County, Kenya.; f Department of Public Health, Masinde Muliro University of Science and Technology, Kakamega County, Kenya.

## Abstract

Maternal and newborn health (MNH) service delivery redesign aims to improve maternal and newborn survival by shifting deliveries from poorly equipped primary care facilities to adequately prepared designated delivery hospitals. We assess the feasibility of such a model in Kakamega County, Kenya, by determining the capacity of hospitals to provide services under the redesigned model and the acceptability of the concept to providers and users. We find many existing system assets to implement redesign, including political will to improve MNH outcomes, a strong base of support among providers and users, and a good geographic spread of facilities to support implementation. There are nonetheless health workforce gaps, infrastructure deficits, and transportation challenges that would need to be addressed ahead of policy rollout. Implementing MNH redesign would require careful planning to limit unintended consequences and rigorous evaluation to assess impact and inform scale-up.

## Abstract (precis)

Service delivery redesign is needed to accelerate progress toward improved health outcomes. Kakamega County, Kenya, demonstrates that there is a strong base of health system assets that would serve as a starting point to successfully implement maternal and newborn health service delivery redesign.

## Teaser

**Key Findings:**

- Maternal and newborn health service delivery redesign (MNH redesign) is a policy to shift all deliveries to or close to hospitals.
- Current system assets that support MNH redesign in Kakamega County are the adequate geographic spread of hospitals, close proximity of women to these hospitals, and high provider and user support for the concept.
- Before MNH redesign is implemented in Kakamega County, prevailing health system deficits, like health provider shortages and transportation challenges for mothers, would need to be addressed.

**Key Implications:**

- The county, in partnership with health system researchers, should rigorously evaluate the process of implementing MNH redesign and its impact on health, to learn and test the model and to serve as a base for generalizing uptake across the country.
- Countries that seek to implement MNH redesign would need to similarly assess feasibility to determine the assets and gaps for implementation.

## INTRODUCTION

Maternal and newborn mortality remain high in many low- and middle-income countries (LMICs), with these countries accounting for more than 90% of the global burden.^1^^,^^2^ At their current rates of decline, most high-burden countries are unlikely to achieve the Sustainable Development Goals (SDGs) for maternal and child health^3^ despite remarkable increases in facility deliveries.^2^ Recent studies demonstrate that giving birth in a facility does not necessarily translate to improved outcomes,^4^^–^^6^ a phenomenon likely attributable to poor quality. More than half of preventable maternal and neonatal deaths in LMICs are estimated to be due to poor quality care during childbirth rather than lack of facility utilization.^7^

Up to 45% of facility deliveries in LMICs occur in primary care facilities.^8^^,^^9^ Many of these primary care facilities lack basic requirements to manage complications arising during delivery, including experienced and specialized staff, supplies, and access to surgical and emergency services, which tend to be available in hospitals.^8^^–^^10^ Primary care facilities may also be located far away from advanced care, with limited referral options.^11^^–^^14^ Thus, improving processes of care in primary care facilities alone is not enough to reduce maternal and newborn mortality.^15^

Against this background, the *Lancet Global Health* Commission on High Quality Health Systems in the SDG Era (the Quality Commission) proposed service delivery redesign, a reorganization of health systems, to optimize outcomes by ensuring that the right care is provided at the right level of the system and by the right provider.^16^ For maternal and newborn health service delivery redesign (MNH redesign), this reorganization means all births would occur in hospitals that provide dignified, patient-centered care and immediate, definitive care for complications (including capacity for cesarean deliveries, blood transfusion, and care for sick mothers and newborns), or in nearby affiliated birthing facilities. In this model, primary care facilities would focus on providing quality antenatal and postnatal care and would be linked to hospitals where women would deliver. In addition, physical access to facilities would be improved through better transport or upgrading primary care centers to support women in remote communities, and communities would be included in the design of the reorganized system. Details about the components of MNH redesign, benefits, potential risks, and key implementation considerations have been previously described.^17^

For MNH redesign, a reorganization of health systems means all births would occur in hospitals that provide patient-centered care and immediate care for complications.

Assessing feasibility is a critical step before embarking on MNH redesign, not only because redesign is a complex health reform but also because its components must be tailored to the specific needs of the local health system and population.

Assessing feasibility is a critical step before embarking on MNH redesign; its components must be tailored to the specific needs of the local health system and population.

In this article, we describe a feasibility assessment conducted in Kakamega County, in western Kenya, to determine the capacity of hospitals to provide services under the redesigned model and the acceptability of the concept to providers and users. Kakamega County is the first setting to embark on service delivery redesign as recommended by the Quality Commission.

## ASSESSMENT PROCESS

### Understanding the Context

Kenya has operated a devolved system of governance since 2010, with 47 semiautonomous counties. Kakamega County is one of the most populous, with a population of approximately 2 million people.^18^ Both the maternal mortality ratio in Kakamega, at 316 per 100,000 live births, and the neonatal mortality rate, at 19 per 1,000 live births, are just below Kenya's average (362 for maternal mortality ratio and 22 for neonatal mortality rate) but well above the SDG targets.^19^^,^^20^ Kakamega County is among the top 15 counties with the highest burden of maternal mortality in the country.^21^ Kenya will miss the SDG targets for mothers and children at the current rates of decline.^3^

According to health management data from Kakamega County's Department of Health, there were 70,084 estimated deliveries in 2018; 35% of these deliveries occurred at home, 28% in primary care facilities (dispensaries (Level 2) and health centers (Level 3)), and 37% in hospitals (Level 4 and 5 facilities). There were 205 facilities in Kakamega County conducting at least 1 delivery in 2018; 58% were very low volume (<52 deliveries per year) and accounted for only 5% of total facility deliveries and 4% were moderate or high volume (>1000 deliveries per year) and accounted for 48% of facility deliveries.

Maternal and newborn care in Kenya is provided free of charge in most facilities through the Free Maternity Care (Linda Mama) program.^22^ Kakamega County has also developed the “Imarisha Afya ya mama na Mtoto" program—popularly referred to as “Oparanyacare”, after the County Governor, Mr. Wycliffe Oparanya, who introduced it—that provides poor and vulnerable women with cash transfers conditional on the use of health facilities for antenatal, delivery, postnatal care and immunization services.^23^

### Stakeholder Consultations

Given the different stakeholders involved in maternal and newborn care, we used a broad and participatory approach for the feasibility assessment. The core study team consisted of individuals from the Kakamega County Department of Health, Kenya Council of Governors, Kenya Ministry of Health, and Harvard University. Consultations were held with health system managers, health care providers, development and implementing partners, health system researchers, and health care users before the start of the assessment to inform its content and strategy. After the analysis stage, we held discussions with the same stakeholder groups to discuss and interpret the findings, identify additional analysis needs, and determine potential strategies for implementation.

### Feasibility Assessment Components

Through consultation, the study team identified 2 broad domains for the feasibility of MNH redesign in Kakamega County: capacity and acceptability. First, the capacity assessment estimated the geographic proximity of women to delivery care, assessed the infrastructure and human resource capacity, and identified potential barriers to care in hospitals. Since the relocation of the place of birth is the biggest change in MNH redesign, these analyses focused on hospital capacity. Second, we aimed to assess the acceptability of MNH redesign among health care providers and health care users.

Determining the feasibility of MNH redesign in Kakamega County involved assessing capacity regarding infrastructure and human resources and assessing acceptability among health care providers and users.

There were 5 major components of data collection and analysis, using a mix of primary and secondary data sources.

1. Geographic analysis: We performed a geographic analysis using the following secondary data: facility geolocations were obtained from the Kenya Master Health Facilities List^24^; distribution of pregnancies came from WorldPop 2015 projection^25^; roads and road classification came from OpenStreetMap;^26^ publicly available shapefiles were used to map out the landcover, road characteristics, and the administrative boundaries of the county. The geographic analysis was conducted using the WHO's AccessMod tool.^27^ We estimated the proportion of pregnancies that were within 2 hours, 1 hour, and 30 mins of a delivery facility (now and under redesign) using motorized transport. Details on the assumptions used in the geographic analysis are included in the Supplement.
2. Facility survey: We conducted a facility survey to assess current facility infrastructure and human resource capacity for maternal and newborn care. Data were collected on administration, infrastructure, health workforce, management and data, clinical services, equipment, materials and supplies, and medicines. All 19 hospitals in the county and a stratified random sample of 30 health centers and dispensaries were selected for inclusion in the survey. This analysis focused on the hospitals since they are the key determinants of the feasibility of the MNH redesign program.
3. Self-administered health care provider survey: We conducted a self-administered health care provider survey to assess knowledge and confidence in the management of key maternal and newborn complications and collect provider perspectives on MNH redesign. A previously validated 60-item survey for testing knowledge on maternal and newborn care formed the base of the knowledge test—recommended pass score: 80%.^28^ The survey was adapted to fit the Kenyan context. All doctors, clinical officers, and nurses/midwives providing maternal and newborn care on the day of the facility survey were eligible to take part in the survey. A total of 151 providers completed the survey of 160 eligible (response rate=94%).
4. Gap analysis: Descriptive statistics were calculated on infrastructure and human resource capacity, and we conducted gap analyses to determine future needs under redesign. The estimated number of deliveries was projected for the year 2021 using average year-on-year increase between 2014 and 2019 and holding subcounty delivery proportions constant (Supplement). We assessed 2 scenarios for each item of interest. The “near-term” scenario assumed that all facility deliveries would shift to the designated hospitals, but no home deliveries are shifted. The “long-term” scenario assumed that all deliveries in the county (facility and home births) would shift to the designated hospitals. These gap analyses were done at the facility level, and the assumptions used in estimating the human resource and other needs were based on international guidelines, Kenyan national guidelines, and consultations with subject matter experts in Kenya^29^^–^^34^ (full list of assumptions included in the Supplement).
5. Focus group discussions (FGDs): We held 16 community FGDs with 119 participants across 4 sites (details included in the Supplement) to explore practical barriers to receiving quality care, opportunities for better health system utilization, and to obtain perceptions on MNH redesign. At each site, 1 FGD was held with each of the following groups: (1) women with a recent home delivery; (2) women with a recent facility delivery and other women of reproductive age who have never delivered; (3) mothers-in-law, grandmothers, and traditional birth attendants/birth companions; and (4) husbands and other male community members. A rapid thematic analysis of the discussion notes was done to summarize the FGD findings.

The feasibility assessment, including initial consultations, data collection and analyses, and final consultations, lasted 7 months, from August 2019 to February 2020. As this was a health system quality improvement project, it was deemed exempt from human subject considerations by the Institutional Ethics Review Board of the Masinde Muliro University of Science and Technology Department of Public Health and by the Harvard University Office of Human Research Administration. All data used for the assessment were deidentified.

## FINDINGS: HOSPITAL CAPACITY IN KAKAMEGA COUNTY

**FIGURE 1:**
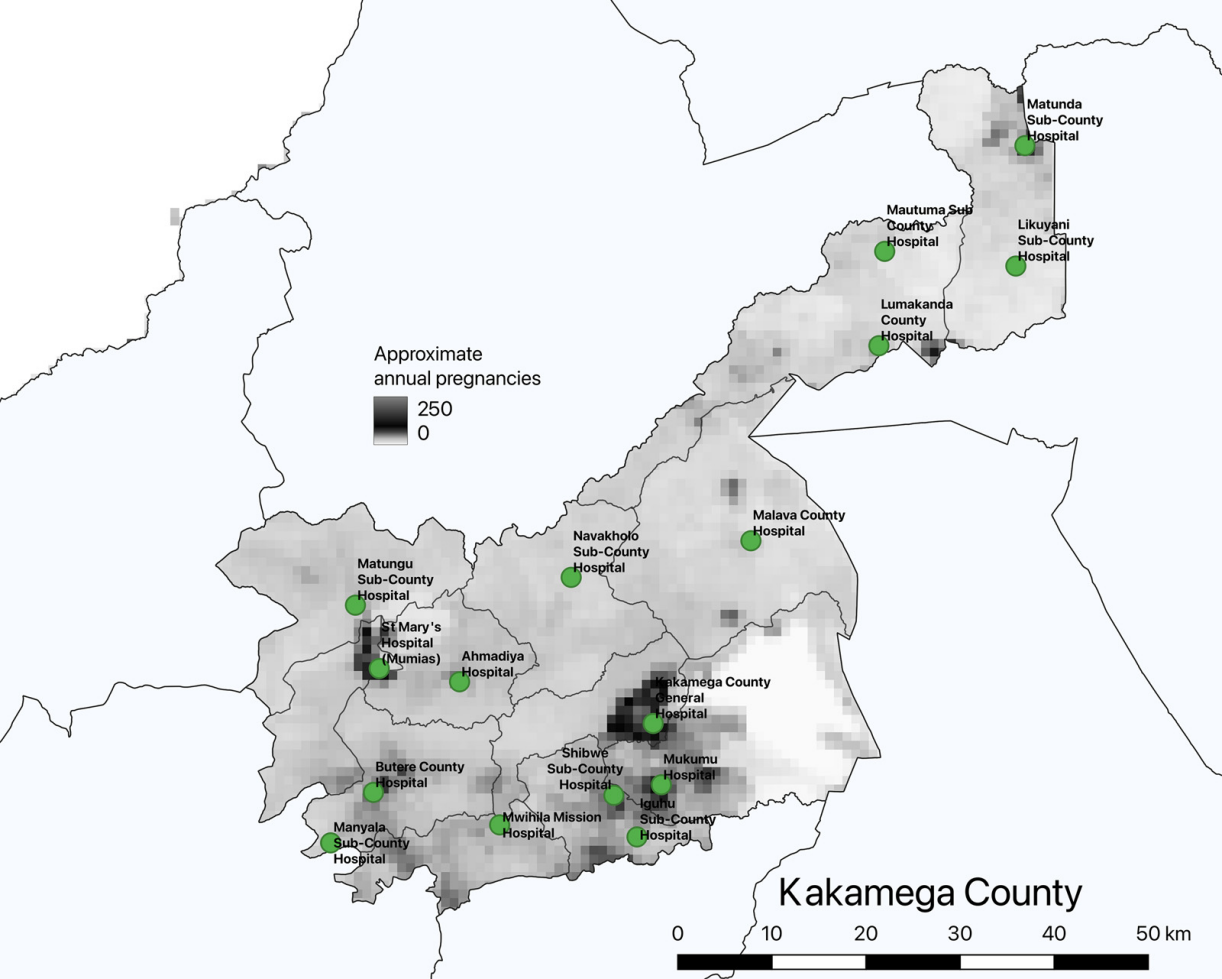
Distribution of 16 Hospitals Identified for Delivery Care With Maternal and Newborn Health Service Delivery Redesign, Kakamega County, Kenya Notes: facility geolocations are from the Kenya Master Health Facilities List^24^ and data on pregnancy densities are from WorldPop (2015).^25^

In consultation with the county health team, the assessment team identified 16 public and faith-based hospitals that could serve as designated delivery hospitals if MNH redesign is implemented in the county. The county decided to focus on these facilities for planning purposes since they already had oversight or contractual relationships with these facilities. As such 3 private for-profit hospitals, along with their 13 surveyed health care providers, were excluded from this initial analysis. However, exclusion of the 3 private for-profit hospitals in the analysis did not significantly change the results. The 16 hospitals together currently conduct more than half of facility deliveries in Kakamega County and are geographically spread among the 12 subcounties (Figure 1).

### Accessibility

The geographic analysis found that all pregnant women (100%) live within 1 hour of travel of current delivery facilities in Kakamega County and 98% live within 30 minutes. Under MNH redesign, 100% of pregnant women would live within 2 hours of travel of a designated hospital, 99% would live within 1 hour, and 85% within 30 minutes.

The FGDs identified transportation availability and cost as barriers to reaching hospitals. Lack of transportation was worse at night when motorbike riders feared to respond to calls due to the risk of being robbed (motorbikes being the most common means of transportation in the county). When transportation was available, the prices were sometimes prohibitive at night: a trip that would typically cost 100 KES (∼US$1) during the day may cost 6 times that at night. A few participants indicated that there was sometimes a preference for home deliveries due to the care and attention provided by traditional birth attendants or for cultural reasons, like the need to bury the placenta in the family home. In these few cases, facility access was limited by cultural and social acceptability rather than by distance, cost, or availability of transportation.

The number of doctors would need to more than triple under the near-term scenario of MNH redesign and the number of nurses/midwives and clinical officers would need to increase 2.5 times to be able to provide care without being overworked.

### Infrastructure

Table 1 displays the existing infrastructure, gaps, and needs under MNH redesign in the 16 designated hospitals. Based on standard occupancy rates and average length of stay (Supplement), we estimated that for the current volume of deliveries in the 16 designated facilities, there are currently 120 excess maternity beds, but that 137 additional beds would be required under the near-term service delivery redesign scenario. The 13 currently functional operating rooms are in 7 of the 16 designated hospitals, and 1 operating room for cesarean deliveries would be required in each of the other designated hospitals under both redesign scenarios. Additional blood transfusion and newborn units would also be required. Additional results on infrastructure are provided in the Supplement.

**TABLE 1.** Current Infrastructural and Human Resource Capacity and Gaps for Redesign Across 16 Designated Hospitals, Kakamega County, Kenya

	Current Available	Current Gap	Gap in Near-Term Redesign Scenario^a^	Gap in Long-Term Redesign Scenario^a^
Infrastructure				
Total maternity beds (including delivery beds)	419	−120^b^	137	457
Functional operating rooms	13	9	9	9
Facilities providing blood transfusion	10	6	6	6
Facilities with functional newborn units	3	13	13	13
Human resources				
Doctors^c^	32	25	110	183
Clinical officers and nurses/midwives	204	183	511	881

a Near-term scenario is the case where deliveries that would have occurred in a facility (45,440 deliveries) are shifted to the 16 designated hospitals, while long-term scenario is the situation in which all deliveries in Kakamega County, both home and facility (72,552 deliveries) are shifted to the 16 redesign facilities. Both scenarios are set in 2021.

b This indicates excess capacity of 120 beds.

c Includes medical officers (general practitioners) and obstetrician/gynecologists.

### Human Resources

There are currently substantial gaps in the number of health care providers (Table 1). The prevailing human resource gap suggests a heavy workload for health care providers, and only 63% of them thought their current workload was manageable. The number of doctors would need to more than triple under the near-term scenario of MNH redesign and the number of nurses/midwives and clinical officers would need to increase 2.5 times to be able to provide care without being overworked. The findings for anesthetists and pediatricians are included in the Supplement.

Beyond the number of staff, we found that health care providers in hospitals had significantly more experience with managing maternal and newborn complications and expressed greater confidence in managing these complications. For example, only 18% of health care providers in primary care facilities reported managing severe pre-eclampsia/eclampsia in the preceding 12 months, compared to 82% of providers in the designated hospitals. However, scores on the knowledge assessment were low across the board. Doctors, who were only found in the hospitals, performed better, with an average score of 68%, albeit still less than the passing score of 80%. Table 2 reports the findings on provider knowledge, experience, and confidence in managing obstetric and newborn complications.

**TABLE 2.** Health Care Provider Knowledge, Experience, and Confidence in Designated Delivery Hospitals and Sampled Primary Care Facilities, Kakamega County, Kenya

	Health Care Providers in Primary Care Facilities^a^(n=65)	Health Care Providers in Designated Hospitals^b^ (n=73)	Comparison (P Value)^c^
Average knowledge scores	54%	57%	.084
Managed complication in past 12 months			
Severe pre-eclampsia/eclampsia	18%	82%	<.001^d^
Post-partum hemorrhage	57%	89%	<.001^d^
Obstructed labor	38%	67%	.001^d^
Newborn resuscitation	62%	89%	<.001^d^
All 4 complications	11%	59%	<.001^d^
Very confident in ability to manage complication			
Severe pre-eclampsia/eclampsia	49%	79%	<.001^d^
Postpartum hemorrhage	77%	92%	.015^d^
Obstructed labor	51%	68%	.034^d^
Newborn resuscitation	71%	74%	.674
All 4 complications	28%	45%	.033^d^

a Health care providers in primary care facilities include 11 clinical officers and 54 nurses/midwives.

b Health care providers in the designated hospitals include 9 doctors, 12 clinical officers, and 52 nurses/midwives.

c A 2-sided student t-test was used for the comparison of the knowledge scores and a chi square test was used for the comparison of all the other variables.

d A *P* value <.05 indicates statistical significance.

## FINDINGS: ACCEPTABILITY OF MNH REDESIGN

Community members (including mothers and other family members) and health workers expressed high support for service delivery redesign.

From the FGDs with community members, the main potential benefit of redesign identified was that delivery in an improved higher-level facility would improve outcomes by reducing the need for referrals. Another potential benefit mentioned was that improving the quality of maternal and newborn care could have a spillover effect on other facility users, e.g., for surgical care. The main challenge identified was transportation because the nearest designated hospital may now be farther away from women. Other concerns were that there may be overcrowding in the designated hospitals, leading to poor-quality care. Many focus group participants identified disrespect, abuse, and lack of patient-centered care as current problems in health facilities, which was sometimes cited as a reason for some mothers preferring to deliver at home (Box).

Community members stated that the main potential benefit of redesign was that delivery in an improved higher-level facility would improve outcomes by reducing the need for referrals.

BOX **Key Participant Quotations in Focus Group Discussions on Maternal and Newborn Service Delivery Redesign in Kakamega, Kenya**

*When you deliver in the hospital you are more “digital”; you are not “analogue”. You are seen to be of high status, and people therefore want to deliver in the facility*. —Mother, aged 36 years, with recent home delivery

*You would have saved us; it would be very good. If this hospital is set up for delivery, then there would be no delays in accessing care*. —Mother, aged 37 years, with a recent home delivery

*If that hospital has everything, then you know that you will not be referred to another facility. Every problem will be managed there. You go to [Level 3 facility], there are no drugs, then you go to [non-surgical Level 4 facility] you cannot be helped and then you are sent to CGH (Level 5 facility), and this could take a very long time*. —Traditional birth attendant, aged 57 years

*Health workers may be a challenge. The health provider may be careless due to overworking*. — Mother, aged 39 years, with recent home delivery

*Some hope to deliver at home, because at home when you are screaming someone would hold you and support you, but in the facility if you scream no one would come and help you. You do not know anyone in the facility and no one is there to support you.* — Mother, aged 28 years, with recent facility delivery

**FIGURE 2:**
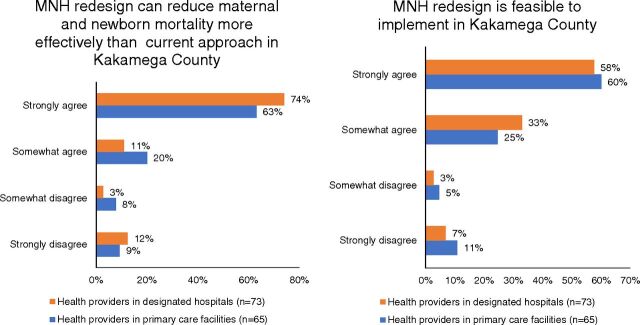
Health Care Provider Perceptions on Maternal and Newborn Health Service Delivery Redesign, Kakamega County, Kenya^a^ Abbreviation: MNH, maternal and newborn health. ^a^Percentages may not add up to 100% due to rounding.

From the health care provider surveys, approximately 85% of respondents either strongly agreed or somewhat agreed that MNH redesign would be more effective in reducing maternal and newborn mortality than the current approach in Kakamega County (Figure 2). Most also believed it would be feasible to implement service delivery redesign in the county.

## INTERPRETING THE FINDINGS

This feasibility assessment found that Kakamega County has a good base of system assets to facilitate a transition to birth in hospitals for all women. There is an adequate distribution of hospitals, health care providers in hospitals demonstrate higher experience with and greater confidence in managing maternal and newborn complications than their counterparts in primary care facilities, and providers and health care users support the idea of MNH redesign. Bottlenecks for MNH redesign include health care provider deficits, health facility infrastructure inadequacies, and transportation challenges.

This assessment shows that hospitals are not distant for most women in Kakamega County. However, there is still a risk of leaving some women behind if concerns about the cost, safety, and ease of reaching a designated hospital are not addressed. Strategies like voucher programs, which are known to increase utilization in similar settings,^35^^–^^37^ could be used to improve access for remote communities. Such a program could be layered on Kakamega's existing conditional cash transfer program for pregnant women, Oparanyacare,^23^ to maximize efficiency and reach.

This assessment shows that hospitals are not distant for most women in Kakamega County, but some women may be excluded from access if concerns about the cost, safety, and ease of reaching a hospital are not addressed.

There is substantial inefficiency in current delivery care in Kakamega County, with the majority (58%) of the 205 delivery facilities each conducting fewer than 1 delivery per week. Aside from quality implications of such low volumes, this represents an inefficient use of scarce health system resources. However, these facilities provide an important primary care function, and antenatal and postnatal care should be enhanced in these facilities as part of the redesign process. The excess of maternity beds suggests other hospital departments may also have similar excesses. If this is the case, a reallocation of these excess beds could reduce the need for new investments with MNH redesign. Beyond this, most hospitals would require an infusion of critical inputs, including the construction of new or improvements to existing operating theaters and newborn units before MNH redesign can be rolled out.

Our assessment found significant health worker deficits, both currently and for MNH redesign. Potential sources of new personnel may be to engage doctors on a part-time basis and reassign some of the nurses/midwives in underused primary care facilities, bearing in mind that some may be performing other functions beyond delivery care. MNH redesign would also result in higher economies of scale for designated hospitals, thus relatively fewer health care providers would be needed.^38^ Based on the low scores observed, provider knowledge and competence would need to be improved. Expert refresher training, specialist supervision, and linkages with obstetric and pediatric training programs may offer a competence boost in the short turn. Since short-term trainings are less durable in sustaining quality and are difficult to scale,^39^ longer-term strategies like reforms to pre-service education would be needed to ensure consistent delivery of quality care.

The FGDs revealed patient concerns about respectful and dignified care in hospitals—a problem that has been highlighted in studies in many LMICs—which is associated with a decreased likelihood of future desire for delivery in the same facility.^40^^–^^42^ Respectful, patient-centered care requires policies, training, and investment ahead of the rollout of MNH redesign to enhance user confidence in the health system.

Two key constituents in the Kakamega health system, health care users and health care providers, demonstrated strong support for MNH redesign. Interestingly, the health care providers at the primary care level were as equally supportive of MNH redesign as their counterparts in the hospitals, irrespective of the fact that the critical, and in some sense prestigious, function of providing birth care would be shifted away from them. This may be due to well-documented stresses of providing delivery care without sufficient backup.^43^

## TAKING A DECISION AND NEXT STEPS

Upon review of the findings of the feasibility assessment, the Kakamega County government has decided to move forward with planning for and implementing MNH redesign. The county plans to roll out the reform in a phased and deliberate manner, starting with 3 of the 12 subcounties. The decision is primarily driven by the county's ambition to improve outcomes for mothers and newborns, as already demonstrated by the county-level initiative, Oparanyacare.^23^ National policies on free maternal care^22^ and the push toward universal health coverage^44^ are also important supportive contextual factors.

Upon review of the findings of the feasibility assessment, the Kakamega County government has decided to move forward with planning for and implementing MNH redesign in a phased manner.

With the decision taken to implement redesign, the next phase of the process is a thorough planning phase. Through this highly participatory planning phase, which includes health system administrators, health care providers, and health care users, and employs a human-centered design approach, the county is outlining the policies, care models, and investments needed to permit the safe rollout of MNH redesign. This includes developing strategies to increase provider numbers and competence within allowed civil service and county budgeting rules and strategies on how to raise funds internally and align development partner funding for the program. Another key consideration at this stage is how the county can ensure that potential risks of this program, including the risk of overmedicalization of births or reducing access for the very remote, can be prevented. Potential strategies that could reduce the risk of overmedicalization include the utilization of midwife-led birthing centers, which are incorporated in or adjacent to designated delivery hospitals. Strategies to address problems with access could include the provision of free transportation, voucher programs, or the establishment of people-centered maternity waiting homes where applicable. With the intended improvement of facilities, another risk is the influx of women from neighboring counties to seek quality birth care in Kakamega county, a situation that can overstretch the infrastructure and health care providers. The county would need to closely monitor delivery volumes when the program is rolled out and prepare a plan to address overutilization. Program costing will also occur at this planning stage; the costing has been completed for the first phase (first 3 subcounties) of implementation. After this planning phase, there will be an improvement phase to address quality, access, and capacity gaps. In this phase, actual health system improvements (in infrastructure, human resource capacity, access, and other sectors) will be undertaken to ensure that the system is ready to support the service delivery redesign program. Only then, after the system has been adequately prepared, would the redesign policy be rolled out and mothers be encouraged to deliver in designated hospitals. There is also a plan for the process of implementation and the impact of redesign to be rigorously evaluated, to inform scale-up outside of Kakamega County.

Another key consideration at the planning stage is how the county can prevent potential risks, including the risk of overmedicalization of births or reducing access for the very remote.

Kakamega County has committed to funding recurring costs of the program and has aligned donor funding for the capital costs of the first phase of implementation. With fiscal space already constrained and the COVID-19 pandemic redirecting funds toward emergency preparedness, inadequate funding may delay the scaleup of redesign. Donor inflows are also dwindling due to the gravitation toward self-reliance in Kenya, which means most of the future infrastructural and human resource improvements would be borne by the county government. However, this is an opportunity for the county to consider the role of the private sector in the provision of health care and to potentially include them in the redesign process as it evolves.

### Limitations

This feasibility assessment had several limitations. First, at the time the feasibility assessment concluded, no costing was included because the price tag of the policy shift depends on the specific solutions the county chooses to address the identified gaps. However, a detailed costing of the first phase of the redesign program has subsequently been completed as part of the planning process to implement redesign. The cost of MNH redesign was of interest to many stakeholders during the consultations at the end of the feasibility assessment and future feasibility assessments should thus consider including some costing for key identified gaps. Second, knowledge assessments and self-administered questionnaires may overestimate skills, and as such, the results of the health care provider assessments need to be considered in the context of mortality and morbidity outcomes and patient-reported outcomes. Where possible, future assessments can consider including observations of service provision to better judge health care provider skills. Lastly, while we conducted a stakeholder assessment with key decision makers in Kakamega County, a broader stakeholder engagement/analysis would be important to better understand the political support for and opposition to redesign.

## CONCLUSION

This feasibility assessment has shown that Kakamega County is ready for MNH redesign: there is political goodwill to improve maternal and newborn health outcomes, a strong base of stakeholder support, and a good spread of facilities to support implementation. There are nonetheless a health workforce gap, infrastructure deficits, and transportation challenges that would need to be addressed ahead of policy rollout.

This feasibility assessment also shows that there is latent capacity in LMICs to institute systems-level change to accelerate progress toward achieving the SDGs. However, redesign is not a one-size-fits-all policy and will look different in different settings. This makes a feasibility assessment a necessary first step. The feasibility assessment methodology presented in this article provides a blueprint for adaptation for countries that seek to embark on MNH redesign.

MNH redesign is not a one-size-fits-all policy; this makes a feasibility assessment a necessary first step in the process.

## Supplementary Material

GHSP-D-20-00684-Supplement.pdf

## Notes

**Peer Reviewed**

**First published online:** November 30, 2021.
