# Supplementary material for: Health System Redesign to Shift to Hospital Delivery for Maternal and Newborn Survival: Feasibility Assessment in Kakamega County, Kenya
Source: Glob Health Sci Pract. 2021 Dec 31;9(4):1000–10. doi: 10.9745/GHSP-D-20-00684 (PMC8691889; doi:10.9745/GHSP-D-20-00684)
Supplement: GHSP-D-20-00684-Supplement.pdf [file GHSP-D-20-00684-Supplement.pdf]

Supplement to: Nimako K, Gage A, Benski C, et al. Health system redesign to shift to hospital delivery for maternal and newborn survival: feasibility assessment in Kakamega County, Kenya. *Glob Health Sci Pract*. 2021;9(4). https://doi.org/10.9745/GHSP-D-20-00684

Supplement

Contents

1. Focus Group Discussion sites and facility survey sample 2
2. Geographic analysis assumptions 3
3. Detailed delivery projections 5
4. Scenario analysis assumptions 6
5. Additional results: Facilities performing key diagnostic tests in previous 3 months among 16 designated hospitals 10
6. Additional results: Facilities with basic equipment and supplies for delivery and newborn care available and functional on day of survey among 16 designated hospitals 11
7. Additional results: Facilities with essential medicines for delivery and newborn care available and unexpired on day of survey among 16 designated hospitals 12

Supplement to: Nimako K, Gage A, Benski C, et al. Health system redesign to shift to hospital delivery for maternal and newborn survival: feasibility assessment in Kakamega County, Kenya. *Glob Health Sci Pract*. 2021;9(4). https://doi.org/10.9745/GHSP-D-20-00684

1. Focus Group Discussion sites and facility survey sample

A total of 16 FGDs were held at four sites spread across the county. The sites were:

1. A peri-urban settlement in Mumias West Sub-county, in the western part of Kakamega County
2. A rural settlement in Lugari Sub-county, in the north-eastern tail of the county
3. A rural settlement in Navakholo Sub-county, in the northern-central part of the county
4. A peri-urban settlement in Shinyalu Sub-county, in the southern part of the county

The distribution of sampled facilities for the facility and provider surveys are shown below:

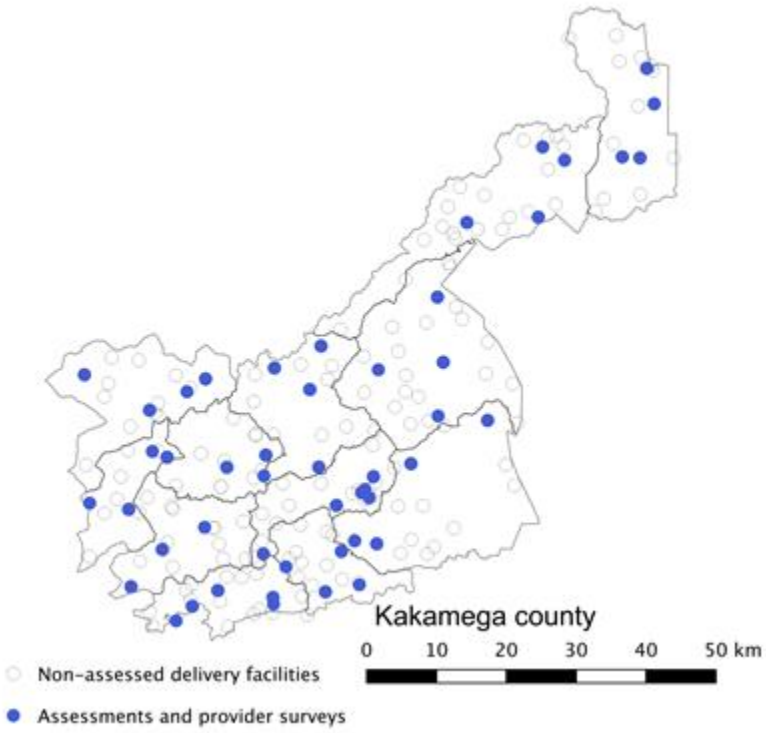

Supplement to: Nimako K, Gage A, Benski C, et al. Health system redesign to shift to hospital delivery for maternal and newborn survival: feasibility assessment in Kakamega County, Kenya. *Glob Health Sci Pract*. 2021;9(4). https://doi.org/10.9745/GHSP-D-20-00684

2. Geographic analysis assumptions

To calculate travel times, we used estimates of walking and vehicular speeds on different road types (speeds used can be found in the supplementary documents). A key assumption made for this analysis was that women would walk to the nearest road and use motorized transport to reach a facility. This analysis does not factor in delays in obtaining a means of transportation nor seasonal changes in road usability. However, one could use the shorter time frames in order to estimate delays that exist within the system.

Speeds used for estimating time to care

**Type of land or road**	**Speed in km/hour**	**Transportation type**
Post-flooding or irrigated croplands (or aquatic)	3	Walking
Rainfed croplands	3	Walking
Mosaic cropland (50-70%) / vegetation (grassland/shrubland/forest) (20-50%)	3	Walking
Mosaic vegetation (grassland/shrubland/forest) (50-70%) / cropland (20-50%)	3	Walking
Closed to open (>15%) broadleaved evergreen or semi-deciduous forest (>5m)	3	Walking
Closed (>40%) broadleaved deciduous forest (>5m)	2	Walking
Open (15-40%) broadleaved deciduous forest/woodland (>5m)	4	Walking
Closed (>40%) needleleaved evergreen forest (>5m)	2	Walking
Open (15-40%) needleleaved deciduous or evergreen forest (>5m)	4	Walking
Closed to open (>15%) mixed broadleaved and needleleaved forest (>5m)	3	Walking
Mosaic forest or shrubland (50-70%) / grassland (20-50%)	4	Walking
Mosaic grassland (50-70%) / forest or shrubland (20-50%)	4	Walking
Closed to open (>15%) (broadleaved or needleleaved, evergreen or deciduous) shrubland (<5m)	3	Walking
Closed to open (>15%) herbaceous vegetation (grassland, savannas or lichens/mosses)	3	Walking
Sparse (<15%) vegetation	5	Walking
Closed to open (>15%) broadleaved forest regularly flooded (semi-permanently or temporarily) - Fresh or brackish water	3	Walking
Closed (>40%) broadleaved forest or shrubland permanently flooded - Saline or brackish water	2	Walking
Closed to open (>15%) grassland or woody vegetation on regularly flooded or waterlogged soil - Fresh, brackish or saline water	3	Walking
Artificial surfaces and associated areas (Urban areas >50%)	5	Walking
Bare areas	5	Walking

Water bodies	0	Walking
Secondary road	40	Motorized
Tertiary road	30	Motorized
Residential road	30	Motorized
Unclassified road	20	Motorized
Trunk road	80	Motorized
Service road	100	Motorized
Steps	4	Walking
Secondary link road	40	Motorized
Tertiary link road	30	Motorized
Path	5	Walking
Primary road	80	Motorized
Pedestrian	5	Walking
Track grade4 road	20	Motorized
Primary Link road	80	Motorized
Trunk link road	100	Motorized
Unknown road	20	Motorized
Track grade2 road	20	Motorized
Track grad3 road	20	Motorized
Track grade5 road	20	Motorized
Track grade1 road	20	Motorized
Cycleway	20	Bicycling
Bridleway	20	Motorized
Living street	20	Motorized

Supplement to: Nimako K, Gage A, Benski C, et al. Health system redesign to shift to hospital delivery for maternal and newborn survival: feasibility assessment in Kakamega County, Kenya. *Glob Health Sci Pract*. 2021;9(4). https://doi.org/10.9745/GHSP-D-20-00684

3. Detailed delivery projections

Sub-county	Facility	Current	Near-term scenario	Long-term scenario
Butere	Butere County Hospital-Level 4	2249	3430	5477
Butere	Manyala Sub-County Hospital-Level 4	403	615	981
Ikolomani	Iguhu Sub-County Hospital-Level 4	855	1240	1979
Ikolomani	Shibwe Sub-County Hospital-Level 4	779	1130	1803
Khwisero	Mwihila Mission Hospital-Level 4	193	1873	2991
Likuyani	Likuyani Sub-County Hospital-Level 4	890	2106	3362
Likuyani	Matunda Sub-County Hospital-Level 4	701	1659	2648
Lugari	Lumakanda County Hospital-Level 4	1764	2987	4770
Lugari	Mautuma Sub-County Hospital-Level 4	647	1096	1749
Lurambi	Kakamega County General Hospital-Level 5	5814	8022	12808
Malava	Malava County Hospital-Level 4	3005	4800	7663
Matungu	Matungu Sub-County Hospital-Level 4	2497	4396	7019
Mumias East	Ahmadiya Hospital-Level 4	331	2099	3351
Mumias West	St Mary's Hospital (Mumias)-Level 4	2489	3794	6058
Navakholo	Navakholo Sub- County Hospital-Level 4	1415	3174	5068
Shinyalu	Mukumu Hospital-Level 4	794	3021	4823
Total		**24826**	**45440**	**72552**

4. Scenario analysis assumptions

Assumptions for 2021 delivery projections

1. The yearly rate of increase for facility deliveries between 2019 and 2021 is 2.9% (this is derived from the average rate of increase per year for facility deliveries over the past 6 years (i.e. 2014 to 2019). See table below.
2. The yearly rate of increase for total deliveries between 2019 and 2021 is 1.0% (this is derived from the average rate of increase per year for total deliveries over the past 6 years (i.e. 2014 to 2019). See table below.
3. The yearly rate of increase in number of pregnancies is the same as the yearly rate of increase in the number of total deliveries. There were an estimated 74283 pregnancies in Kakamega in 2018

**Period**	**Estimated county deliveries (KNBS)**	**Health facility deliveries (KHIS)**
2014	68015	38222
2015	71321	42171
2016	72756	43095
2017	69134	37987
2018	70084	45339
2019	71122	42914
2020- projection	71834	44159
2021- projection	72552	45440

KNBS- Kenya National Bureau of Statistics
KHIS- Kenya Health Information System

Assumptions for gap analysis for needed maternity beds

Needed maternity beds calculated with the formula:

$$\frac{\left[\left(N_{vaginal} * ALOS_{vaginal}\right) + \left(N_{csection} * ALOS_{csection}\right)\right] * Inflationfactor}{365 * Occupancy\ rate}$$

With the following assumptions:

1. Bed occupancy rate: 80% (target from Kenya Health Facility Assessment, 2018)[28]
2. Average length of stay (ALOS) for vaginal delivery = 2 days (12 hours of labor, 24 hours of postnatal observation, 12 hours for bed clean-up and changeover)
3. Average length of stay (ALOS) for Cesarean section (C/S) = 4 days (12 hours of labor, 3 days of postnatal observation, 12 hours for bed clean-up and changeover)
4. Inflation factor of 1.454 accounts for beds in antenatal ward and for long-stay postnatal patients, e.g. for KMC (derived from the current proportion of maternity beds dedicated to ANC and KMC in the 8 facilities reporting these numbers during primary data collection)
5. Cesarean section rate in future scenario is 18% (this is the current rate of C/S in the public and faith-based surgical facilities in Kakamega)

6. Delivery volume in each facility in future scenarios is proportionate to sub-county proportion of deliveries
7. Deliveries by women from outside Kakamega County in facilities in Kakamega County is balanced out by delivery by Kakamega County residents in facilities outside Kakamega County
8. Level 5 facility will attend to some cases that are more complicated and require extended lengths of stay. Additional beds were added to the Level 5 facility based on the assumption that 1% of all pregnancies in Kakamega would develop a complication that would require extended stay for care (i.e. double the average length of stay in the antenatal ward) in a Level 5 facility. The rate of severe maternal morbidity in population-based samples in Africa is 6-7%[29], but most of such cases can be managed in lower facilities (Level 4), hence the estimate of 1% chosen for severe cases that would be referred to the Level 5 facility.

Assumptions for functional operating rooms

The Kenya National Health Infrastructure Norms and Standards (2017) recommends one maternity theatre for each facility, independent of the facility's level.[30] This is reasonable since the theatre can perform nearly 3000 operations in a year if operating 24 hours a day and year-round (a conservative estimate of 3 hours for each operation, including operating time and time for cleaning and set up, would allow 2920 surgeries to be performed in the theatre per year). According to our projections, the busiest facility when all deliveries in Kakamega are occurring in the 16 redesign facilities would be doing approximately 13000 total deliveries and 2300 Cesarean sections in a year.

Assumptions for human resources gap analysis: Doctors (Medical officers and OB/GYNs), nurses/midwives and clinical officers for obstetric care

Needed doctors calculated with the formula:

$$\frac{[doctors\ per\ shift \mid FIGO\ statement] \ast 24 \ast 365}{hours\ of\ work_{year}}$$

Needed nurses/midwives and clinical officers (COs) calculated with the formula:

Nurses/midwives and COs required for obstetric= nurses/midwives needed for delivery care + nurses/midwives needed for care in antenatal ward + nurses/midwives needed for care in postnatal ward:

Nurses/midwives and COs for delivery care:

$$\frac{[nurses\ per\ shift \mid FIGO\ statement] \ast 24 \ast 365}{hours\ of\ work_{year}}$$

Nurses/midwives and COs for antenatal ward:

$$\frac{N_{ancward\ admissions} * ALOS_{ancward} * 24}{hours\ of\ work_{year} * Patients\ \ per\ staff}$$

Nurses/midwives and COs for postnatal ward:

$$\frac{[(N_{vaginal}\ *) * ALOS_{pnc\_vaginal}]\ +\ [(N_{csection}) * ALOS_{pnc\_C/S}] * 24}{hours\ of\ work_{year} * Patients\ \ per\ staff}$$

With the following assumptions:

1. Minimum staffing standards for delivery care (for women in labor and immediate postpartum) by FIGO was used to calculate surgical and non-surgical staff required for delivery care[37]
2. All medical officers (GPs) can perform Cesarean sections
3. Nurses/midwives and clinical officers perform similar functions
4. All health personnel work full-time in the delivery unit
5. Total working hours per employee/year: 1656 (calculated based on a 40-hr working week and 53 working days of leave and absences (21 days annual leave, 7 days sick leave, 13 days public holidays and 12 days other leave days- training, maternity leave, etc.)
6. Patient:staff ratio for antenatal and postnatal ward care (for nurses/midwives and clinical officers): 8:1 (This ratio is recommended by FIGO for the monitoring of women in the latent phase of labor or who are being induced. This ratio was thus chosen for the antenatal and postnatal care wards since it was assumed that the workload in these areas would be similar to monitoring for the latent phase/induction)
7. Average length of stay after vaginal delivery= 1 day
8. Average length of stay after Cesarean section= 3 days
9. Average length of stay in antenatal ward= 5 days (average length of stay of 5 days was chosen after consultation with a nurse-midwife in Kakamega county)
10. Proportion of ANC admissions to total admissions in delivery unit = 0.15 (This is the current ratio among the 46 facilities providing the relevant data during primary data collection).

Assumptions for human resources gap analysis: Nurses/midwives and clinical officers required for newborn care

Needed nurses/midwives and clinical officers (COs) calculated with the formula:

$$\frac{N_{newborn\ admissions} * ALOS_{NBU} * 24}{hours\ of\ work_{year} * Patients\ \ per\ staff}$$

With the following assumptions:

1. Proportion of live births requiring admission= 18.3%. This is the estimate suggested by Murphy and colleagues for Nairobi City[31]

Assumptions 2-13 are from work by Tsiachristas and colleagues[32]

2. Proportion of admitted newborns requiring standard care= 74%
3. Proportion of admitted newborns requiring intermediate care= 13%
4. Proportion of admitted newborns requiring intensive care= 13%

5. Average length of stay (ALOS) in intensive care newborn unit (NBU) after admitted to intensive care= 3.0 days
6. ALOS in intermediate care NBU after admitted to intensive care NBU= 2.0 days
7. ALOS in standard care NBU after admitted to intensive care NBU= 1.0 day
8. ALOS in intermediate care NBU after admitted to intermediate care NBU= 3.0 days
9. ALOS in standard care NBU after admitted to intermediate care NBU= 2.0 days
10. ALOS in standard care NBU after admitted to standard care NBU= 3.5 days
11. Number of sick newborns per staff in standard care= 6
12. Number of sick newborns per staff in intermediate care= 3
13. Number of sick newborns per staff in intensive care= 1
14. Standard and intermediate care can occur at either Level 4 or 5 facilities, and intensive care will only occur at the Level 5 facility
15. Number of livebirths was estimated from the ratio of current facility deliveries to livebirths in the primary data collected. A ratio of 0.9885 was estimated from the dataset. This ratio is similar to that obtained from the DHIS-2 data for all facility deliveries, i.e. live births of 44530 and total deliveries of 45339, giving a ratio of 0.98216. We used the estimate from the dataset because it is more conservative. It is also more conservative than calculating it from the crude birth rate and population projection as has been done in previous studies by Murphy and colleagues.[33]
16. All personnel work full-time in the newborn unit

Assumptions for human resources gap analysis: Anesthetists required for surgical care and pediatricians required for newborn care

We did not find any published standards based on volume of care for anesthetists or pediatricians. For anesthetists, we assumed a 1:1 patient to provider ratio and 6 hours of care per patient. Using the number of Cesarean sections per facility, we estimated that 15 full-time equivalents are currently required to provide obstetric anesthesia across the facilities that are providing obstetric surgical care, while 38 are required in the near-term redesign scenario and 58 in the long-term redesign scenario. 31 anesthetists are currently available in these facilities for all services (not just obstetric care).

For pediatricians required for newborn care, we recommend a minimum of one each per facility, and therefore a total minimum of 16 would be needed now and with redesign. There are currently only 4 pediatricians in the 16 designated facilities.

Supplement to: Nimako K, Gage A, Benski C, et al. Health system redesign to shift to hospital delivery for maternal and newborn survival: feasibility assessment in Kakamega County, Kenya. *Glob Health Sci Pract*. 2021;9(4). https://doi.org/10.9745/GHSP-D-20-00684

5. Additional results: Facilities performing key diagnostic tests in previous 3 months among 16 designated hospitals

	Test	N	%
1	HIV test	16	100
2	Syphilis test (rapid or VDRL)	16	100
3	Malaria rapid diagnostic test or microscopy	16	100
4	Urine rapid tests for pregnancy	16	100
5	Urine dipstick for protein	16	100
6	Rapid test for glucose	16	100
7	Hemoglobin test	16	100
8	Blood typing (ABO blood group test and Rhesus blood group test)	16	100
9	Cross match testing	10	63
10	Clotting profile	2	13
11	Renal (kidney) function test	5	31
12	Liver function test	4	25
13	Serum bilirubin test	6	38

6. Additional results: Facilities with basic equipment and supplies for delivery and newborn care available and functional on day of survey among 16 designated hospitals

	Item	N	%
1	Examination light	14	88
2	Delivery pack (including a cord clamp, episiotomy scissors, scissors/blade to cut cord, suture material with needle, AND needle holder)	16	100
3	Suction apparatus (suction bulb or electric suction pump)	16	100
4	Manual vacuum extractor	15	94
5	Vacuum aspirator or D&C kit (with speculum)	13	81
6	Resuscitator bag and mask- adult	14	88
7	Neonatal bag and mask size 1- for term babies	16	100
8	Neonatal bag and mask size 0- for pre-term babies	14	88
9	Resuscitaire or Resuscitation table with heat source for newborn resuscitation	14	88
10	Blank partograph	16	100
11	Sterile gloves	16	100
12	Infant weighing scale in delivery area	16	100
13	Blood pressure apparatus in delivery area	14	88
14	Pulse oximeter- adult	8	50
15	Pulse oximeter- neonatal	2	13
16	Phototherapy unit	8	50
17	Oxygen (delivered through a cylinder, from a central supply or by oxygen concentrators)	15	94

Supplement to: Nimako K, Gage A, Benski C, et al. Health system redesign to shift to hospital delivery for maternal and newborn survival: feasibility assessment in Kakamega County, Kenya. *Glob Health Sci Pract*. 2021;9(4). https://doi.org/10.9745/GHSP-D-20-00684

7. Additional results: Facilities with essential medicines for delivery and newborn care available and unexpired on day of survey among 16 designated hospitals

	Medicine	N	%
1	Oxytocin (injectable)	16	100
2	Misoprostol (cap/tab)	12	75
3	Sodium Chloride (Saline Solution) (injectable solution)	16	100
4	Azithromycin (cap/tab or oral liquid)	12	75
5	Calcium gluconate (injectable)	15	94
6	Cefixime (cap/tab)	11	69
7	Magnesium sulphate (injectable)	16	100
8	Benzathine benzylpenicillin powder (for injection)	15	94
9	Ampicillin powder (for injection)	7	44
10	Betamethasone or Dexamethasone (injectable)	14	88
11	Gentamicin (injectable)	15	94
12	Nifedipine (cap/tab)	16	100
13	Metronidazole (injectable)	13	81
14	Hydralazine	16	100
15	Antibiotic eye ointment for newborn	15	94
